# Renal Sugar Metabolites and mRNA Expression of Glucose Transporters in Meat-Type Chickens with Differing Residual Water Intake

**DOI:** 10.3390/ani14192912

**Published:** 2024-10-09

**Authors:** Marie C. Milfort, Ahmed F. A. Ghareeb, Oluwatomide W. Ariyo, Josephine Kwakye, Evan Hartono, Selorm Sovi, Bikash Aryal, Alberta L. Fuller, Mohamed I. El Sabry, Farid Stino, Romdhane Rekaya, Samuel E. Aggrey

**Affiliations:** 1 Water Intake Genomics Laboratory, Department of Poultry Science, University of Georgia, Athens, GA 30602, USA; milfort@uga.edu (M.C.M.); ahmed.ghareeb@uga.edu (A.F.A.G.); oluwatomide.ariyo@uga.edu (O.W.A.); josephine.kwakye@uga.edu (J.K.); evan.hartono@uga.edu (E.H.); selorm.sovi@uga.edu (S.S.); bikash.aryal@uga.edu (B.A.); alfuller@uga.edu (A.L.F.); 2 Department of Animal Production, Cairo University, Giza 12613, Egypt; m.elsabry@gmail.com (M.I.E.S.); farid.stino@gmail.com (F.S.); 3 Department of Animal and Dairy Science, University of Georgia, Athens, GA 30602, USA; rrekaya@uga.edu

**Keywords:** water intake, kidneys, glucose transporters, broilers, residual water intake

## Abstract

**Simple Summary:**

It is vitally important that methods to combat climate change-induced effects on water availability are studied in agriculture. We have previously determined the voluntary water usage of modern broilers and, in this article, further explore the molecular differences between meat-type chickens that consume more or less water than average. We show here that sugar metabolites and the expression of glucose transporter genes in the kidneys are different between these birds. These differences can aid in the selection of birds that drink less water than average for the same feed intake and body weight gain. This could help reduce the use of water in the poultry industry.

**Abstract:**

Molecular differences exist between birds with high residual water intake (HRWI) compared to those with low residual water intake (LRWI). Residual water intake (RWI) is defined as the difference between the water intake of a bird and the expected water intake corrected for metabolic body weight, feed intake, and body weight gain. Tissue metabolomic analysis revealed significantly increased kidney glucose, fructose, and arabitol in the LRWI group compared to the HRWI group. mRNA expression analysis of apical sodium glucose cotransporters SGLT1, SGLT4, SGLT5, and SGLT6 showed decreased expression of SGLTs 1, 5, and 6 in LRWI birds (*p* < 0.05), whereas SGLT4 expression was increased compared with HRWI birds (*p* < 0.01). An analysis of basal glucose transporters GLUT1, GLUT2, GLUT5, and GLUT9 showed significantly increased GLUT2 expression in LRWI birds compared with HRWI birds (*p* < 0.01). We postulate that SGLT4 is the main apical transporter in chicken kidneys and that its increased expression reduces these birds’ need for water, resulting in less drinking. This is balanced by the increased expression of the basal transporter GLUT2, indicating better glucose retention, which may partly explain the physiological mechanism behind why these birds drink less water. Innately driven broiler water intake could therefore be influenced by the expression of kidney solute transporters.

## 1. Introduction

Water scarcity, exacerbated by climate change, poses a significant challenge to the agricultural industry and innovative strategies are needed to combat this issue and encourage sustainable practices. In their 2023 World Water Development Report, the UN reported that 72% of freshwater use is for agriculture, which is increasingly in competition with urban water demand [1]. The World Bank recommends policy interventions to improve water efficiency and the resilience of our food systems to climate shock-induced water threats [2]. Much research has been focused on water metabolism in humans, plants, and select agricultural animals, but data on the molecular mechanisms that underlie voluntary water intake in chickens, a globally important protein source, remain scant.

World chicken production and consumption have increased considerably within a few decades [3]. As with the rest of the economy, chicken production growth was affected by COVID-19, followed by avian influenza, but it remains the most consumed meat product in the world and global consumption is projected to grow by almost 17% over the next decade [3]. As the world population is expected to grow by 0.9% per year for the next decade, one can expect the increased demand for poultry products to increase competition for water resource allocation between agriculture and human use [3]. Therefore, it is important to research methods by which we may increase our resilience to water stress before the inevitable clash over water resources. In addition to smart water management, one method at our disposal is to select farm animals that consume less water for the same performance.

We previously showed that birds that consume less water on average yet provide the same production parameters have significant molecular differences to birds that drink more water [4]. We characterized birds that consume more than the average expected for their body size as having high residual water intake (HRWI) and birds that consume less than the average expected for their body size as having low residual water intake (LRWI). We reported that HRWI and LRWI birds consumed an average of 3.4 kg of feed, but 9.1 kg of water for HRWI birds and 6.9 kg of water for LRWI birds. We showed that both LRWI and HRWI birds had significant differences in their expression of genes important in water regulation and transport in the hypothalamus and the kidney [4]. Specifically, birds with LRWI had significantly higher hypothalamic expression of arginine vasopressin and kidney aquaporin 2, which are essential for the reabsorption of water.

There are significant differences between chicken kidneys and human kidneys [5]. These differences are not only anatomical but are also present at the molecular level. Grossly, chicken kidneys contain both mammalian-type and reptilian-type nephrons, with the latter being more numerous [6]. The anatomical and molecular differences result in the production of uric acid as a waste product instead of urea [7]. Furthermore, chickens are reported to be in a hyperglycemic state compared to mammals [8]. The blood glucose levels of birds can be as high as four times those of mammals but without the associated pathologies. This difference may be true also in regard to tissues and requires further inquiry into glucose metabolism in chicken kidneys. In fact, higher tissue glucose has been shown in birds living at higher elevations and colder temperatures; thus, stress may also lead to high glucose in tissues [9]. But the high glucose levels in the blood of birds are not coupled to an increase in oxidative stress as they are in mammals; therefore, they are adapted to higher glucose without shortening their lifespan relatively [10].

In the mammalian kidney, glucose transport is achieved via the GLUT and SGLT protein families for passive and active transport, respectively, with SGLT1 and 2 on the apical membranes and GLUT2 on the basolateral membrane [11,12]. Avian glucose transporters are not as well studied and public nucleotide database searches reveal that chickens do not have an avian counterpart to SGLT2, which is responsible for most of the glucose reabsorption into renal proximal tubule cells in mammals. This begs the question whether SGLT1 replaces the function or whether one of the other SGLTs is functioning in place of SGLT2 in chicken kidneys.

Our objective herein was to examine the differences between HRWI and LRWI birds in their transport of glucose in the kidney to further characterize the molecular basis of their differences in water intake. This can aid in efforts to improve the water utilization efficiency of broilers and, therefore, the resilience of the industry to water shocks.

## 2. Materials and Methods

### 2.1. Ethics Statement

The chickens used in this experiment were raised at the Poultry Research Center at the University of Georgia according to the regulations of its Institutional Animal Care and Use Committee. All experiments in this study were performed under Animal Use Proposal (AUP) number A2021 07-003-Y1-A0 approved by the Animal Care and Use Committee (IACUC) of the University of Georgia.

### 2.2. Birds

Seven hundred and twenty Ross 708 broiler chickens were raised in colony cages (3.1 m × 1.2 m) for 2 weeks, following which they were placed in individual cages (L = 30.48 cm, W = 45.72 cm, H = 60.96 cm). Equal numbers of males and females were placed. Birds were managed following standard animal care practice. Each bird had its own feeder and drinker for individual measurements. On day 14, initial feeder and drinker weights were measured and the differences in weights on days 21, 28, 35, and 42 were used to calculate intake. Weekly water and feed intakes were measured for each individual bird on days 21, 28, 35, and 42. The room temperature and relative humidity were maintained at the thermal comfort zone following the Ross 708 management manual [13]. A recommended grower diet was fed from 14 to 28 days, and a finisher diet was fed from 28 to 42 days [13].

### 2.3. Sampling

On day 42, 7 males with the lowest RWI (average = −1.2 L) and designated LRWI and 7 males with the highest RWI (average = +1.5 L) and designated HRWI were selected and humanely euthanized by cervical dislocation. Kidneys were removed and placed in liquid nitrogen and later stored at −80 °C. Details of the determination of RWI have been described by Aggrey et al. [4].

### 2.4. Sugar Metabolites

One hundred milligrams of kidney tissue were analyzed for metabolomic profiles by Metabolon Inc. (NC, USA). Briefly, samples were extracted, deproteinated, dried, reconstituted, and analyzed by mass spectrometry with several controls. Methods used on four separate fractions included ultrahigh performance liquid chromatography–tandem mass spectroscopy (UPLC-MS/MS) using acidic positive ion conditions optimized for (1) hydrophilic or (2) hydrophobic compounds, (3) basic negative ion conditions, and (4) negative ionization. The MS scan range was between 70 and 100 mass-to-charge ratios (*m*/*z*). Detailed metabolomic procedures are reported by Aggrey et al. [14]. Metabolite identification and quality control were performed using the Metabolon laboratory information management system (Metabolon Inc., Research Triangle Park, NC, USA). The data were analyzed using ArrayStudio v 10.0. Welch’s two-sample *t* test was used to separate the means, which was executed in ArrayStudio/Jupyter.

### 2.5. RNA Extraction, cDNA Synthesis, and RT-qPCR

Tissue RNA was extracted from 100 mg of kidney tissue following the procedural guidelines of the TRIzol reagent (Invitrogen, Waltham, MA, USA cat # 15596018). The eluate was then cleaned up using an RNeasy Mini Kit following manufacturer protocols (Qiagen, Hilden, Germany cat # 74106). RNA concentration and purity were evaluated using a NanoDrop 2000 spectrophotometer (Thermo Scientific, Waltham, MA, USA) and diluted to 200 ng/uL. Ten microliters of the diluted RNA was used as template according to the protocols of a High-Capacity cDNA Reverse Transcription Kit (Applied biosystems, Foster City, CA, USA cat # 4368813) to produce cDNA using Gradient Mastercycler (Eppendorf, Hauppauge, NY, USA) adjusted for the following cycles: 10 min at 25 °C, 120 min at 37 °C, 5 min at 85 °C, and a final cycle at 4 °C. The synthesized cDNA was quantified using a nanodrop spectrophotometer and subsequently diluted to 20 ng/uL. Two microliters of the diluted cDNA were used with 0.6 uL of forward and reverse primers as indicated in the primer table below (Table 1), with 6.8 uL of molecular water and 10 uL of PowerUp SYBR Green for each qPCR reaction (Applied Biosystems cat # A25778). Quantitative PCR was performed using StepOnePlus (Applied Biosystems, Foster City, CA, USA) with program settings 50 °C for 2 min, 95 °C for 2 min, 40 cycles of 95 °C for 15 s, and 60 °C for 1 min. Each biological sample was run in triplicate. The Ct values at the endpoint and the melting temperature curve for each endpoint were measured. The Ct values of the genes of interest were normalized against the Ct values of the β-actin gene (endogenous control), and the fold change in the LRWI was calculated relative to the HRWI group.

Relative mRNA expression was computed with the 2-ΔΔCt method [15] for glucose transporter type GLUT1 (SLC2A1), GLUT2 (SLC2A2), GLUT5 (SLC2A5), and GLUT9 (SLC2A9) and sodium-dependent glucose cotransporter SGLT1 (SLC5A1), SGLT4 (SLC5A9), SGLT5 (SLC5A10), and SGLT6 (SLC5A11). Statistical significance in relative gene expression between LRWI and HRWI for the genes was calculated using PROC GLM in SAS with a significance level of *p* < 0.05 [16].

## 3. Results

**Figure 1:**
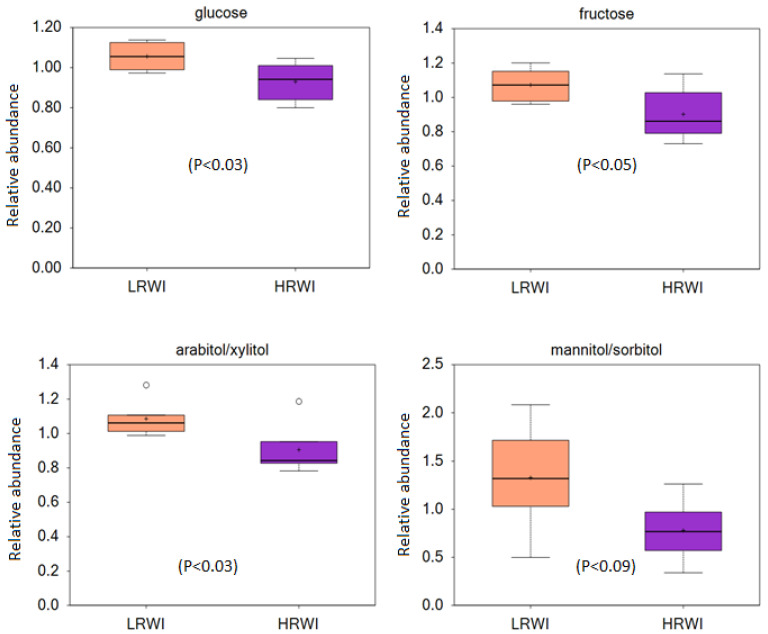
Boxplot of scaled concentrations of kidney glucose, fructose, arabitol/xylitol, and mannitol/sorbitol metabolite differences in birds with high (HRWI) and low residual water intake (LRWI). Y axis is represented as scaled units. Data were normalized to the total spectral area.

The differences in glucose, fructose, arabitol/xylitol, and mannitol/sorbitol among the two chicken groups are shown in Figure 1. The statistical analysis revealed that kidney glucose, fructose, and arabitol/xylitol levels were significantly higher (*p* < 0.05) in the LRWI group compared with the HRWI group. The kidney level of mannitol/sorbitol followed the same pattern, showing higher concentration in the LRWI chickens compared to the HRWI chickens (Figure 1). However, the difference was not significant (*p* < 0.09).

**Figure 2:**
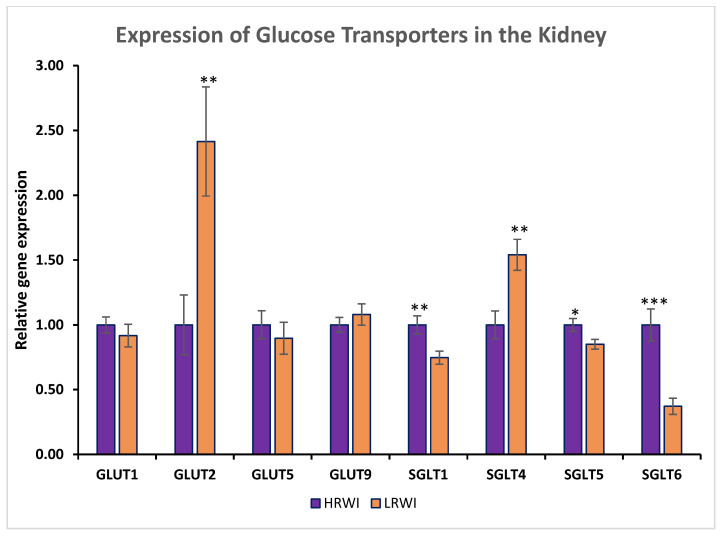
mRNA expression of glucose transporters GLUT1, GLUT2, GLUT5, GLUT9, SGLT1, SGLT4, SGLT5, and SGLT6 in the kidneys of birds with high residual water intake (HRWI) and low residual water intake (LRWI) (* *p* < 0.05, ** *p* < 0.01, *** *p* < 0.001).

**Figure 3:**
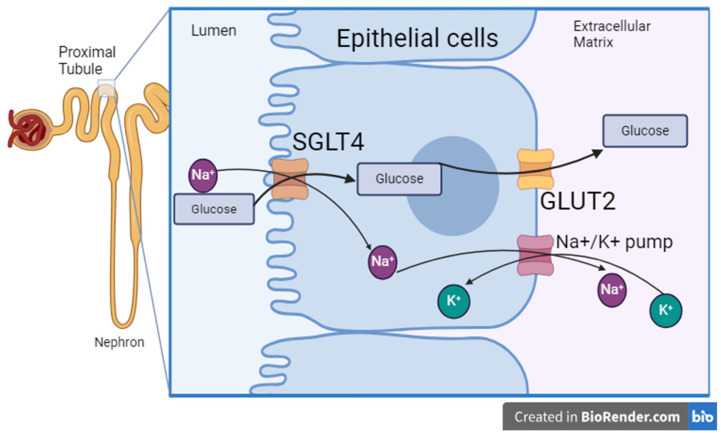
Model describing the mechanism by which glucose is transported across the proximal tubular epithelium in chicken kidneys. SGLT4: sodium glucose transporter 4, solute carrier family 5 member 9 (SLC5A9); GLUT2: glucose transporter type 2, solute carrier family 2 member 2 (SLC2A2).

The relative gene expression values of basal glucose transporters GLUT1, GLUT2, GLUT5, and GLUT9 and sodium-dependent glucose transporters SGLT1, SGLT4, SGLT5, and SGLT6 in the kidneys of HRWI and LRWI birds are presented in Figure 2. There was no significant difference between the kidney expression levels of GLUT1, GLUT5, and GLUT9 in the two investigated groups, LRWI and HRWI. Interestingly, the expression levels of GLUT2 were significantly higher (*p* < 0.01) in chickens with LRWI compared to those with HRWI. For the apical transporters, SGLT1, SGLT5, and SGLT6 were downwardly expressed in chickens with LRWI compared to chickens with HRWI (*p* < 0.01, 0.05, and 0.001, respectively). However, SGLT4 was significantly upwardly expressed in LRWI birds versus HRWI birds (*p* < 0.01). Based on the results, we surmise that mammalian SGLT2’s function could be carried out by SGLT4 in birds (Figure 3).

## 4. Discussion

In humans, hyperglycemia causes water movement out of cells and into the serum which dilutes sodium concentrations [17]. Chickens have a basal blood glucose level that would be considered hyperglycemic in the majority of mammals and are reported to be insulin resistant [8]. Interestingly, those studies often used insulin from other species (bovine, porcine, human) which may be structurally different enough to chicken insulin to provide the appearance of resistance necessitating higher doses for action [18,19,20]. In fact, chicken insulin is reported to be more potent than porcine insulin and elicits the normal expected response of lowering blood glucose at physiological plasma levels in chickens [21]. Thus, the comparative hyperglycemia seen in chickens may also be present in tissues and affect water movement.

The glucose, fructose and arabitol levels were higher in the kidneys of birds that require less water for the feed intake and weight gain compared to their counterparts that consumed significantly more water for similar feed intake and weight gain. Higher sugars in tissues may help them retain more water for osmotic balance. Arabitol is not well studied in chickens. Its concentration in the serum has been shown to be associated with woody breast myopathy and it is elevated in fecal contents when probiotics are administered [22,23]. It can be produced by microorganisms from glycerol, glucose, or arabinose but its synthesis and function in kidney is unknown and it may simply be another sugar that is retained more in LRWI birds [24]. Based on their higher kidney glucose levels, we intimated that glucose transport in the kidneys may be different in LRWI birds compared with HRWI birds. We therefore investigated the relative mRNA expressions of various glucose transporters in these two types of broilers.

GLUT1 is a transmembrane protein that facilitates the diffusion of glucose across a membrane. It is responsible for basolateral glucose uptake and is expressed in almost all cell types [25]. In the kidney, GLUT1 is expressed in both the glomerular and tubular compartments [26]. There was no significant difference between the two groups in kidney GLUT1 expression. This may be because glucose transport through GLUT1 is dependent on a low glucose concentration in the receiving compartment and may not be the main transporter in use in the kidney. GLUT1 cannot move glucose against its concentration gradient from a region of low glucose concentration to a region of high glucose concentration [25]. Therefore, it is not the transporter most responsible for transporting glucose from the kidney lumen into the cells against its concentration gradient, and a change in its expression would not necessarily affect glucose transport.

Current understandings of mammalian kidney glucose reabsorption identify GLUT2 as the basolateral membrane passive transporter for glucose exiting proximal tubule cells and entering the plasma [27]. Renal GLUT2 is increased in diabetes, increasing glucose reabsorption and the loss of kidney GLUT2, reversed hyperglycemia, and normalized body weight in a mouse model of diabetes and obesity [28]. The increased mRNA expression of GLUT2 in LRWI birds may be the cause of the increased glucose concentrations observed in the kidneys of LRWI birds. This local hyperglycemia at the tissue level may result in water movement into the tissue, which could putatively explain why the water intake in the LRWI group is significantly lower compared to that of the HRWI chickens. It has been shown that GLUT1 protein and mRNA steady-state levels were reduced and GLUT2 protein and mRNA levels were increased in streptozotocin-induced diabetic rats [29]. However, whether the increased absorption of glucose causes the reduced water intake or the reduced water intake causes the increased glucose reabsorption is not clear at this time and will require further exploration.

GLUT5 is present at the apical plasma membrane of the proximate tubule cells and serves as a fructose transporter. Fructose is an important component for the formation of advanced glycan end products in diabetes [30,31]. There is an indication that the main role of GLUT 9 is not glucose transportation [32]. Preitner et al. showed that in the kidney, GLUT9 sustains urate reabsorption independent of other known urate transporters, URAT1, OAT1, and OAT3 [33]. The mRNA expressions of GLUT5 and GLUT9 were not significant to water intake in chickens as there were no differences in expression between the two groups. But we do report their expression in chicken kidney as has been previously reported for GLUT9 [34,35]. In chickens, GLUT9 is abundantly expressed in the jejunum and ileum, and a report by Ding et al. suggests that GLUT9 in chicken liver and kidney regulates serum uric acid [35]. GLUT1, GLUT5, and GLUT9 do not show significant differences, likely because their contribution to glucose transport and water retention in the avian kidney is trivial.

The sodium–glucose cotransporters are responsible for the tubular reabsorption of glucose from the kidney lumen into the tubular cells. In mammals, SGLT2 is a sodium-dependent glucose transport protein. SGLT2 is the major cotransporter involved in glucose reabsorption in the kidney. SGLT2 is located in the proximal tubule and is responsible for the reabsorption of 80–90% of the glucose filtered by the kidney glomerulus in rodents and humans [36,37,38]. Most of the remaining glucose absorption is by SGLT1 in more distal parts of the proximal tubule [39]. SGLT4 is also reported to transport glucose but has a higher affinity for mannose and is therefore assumed to have a primary role in mannose transport [38,40]. Human SGLT5 has been shown to be associated with renal fructose reabsorption and hepatic lipid metabolism [41]. Human SGLT6, also known as sodium-myo-inositol transporter 2 (SMIT2), has been reported to be an active transporter for myo-inositol and D-glucose [42].

In the current study, SGLTs 1, 5, and 6 show decreased expression in the kidneys of LRWI birds. The significance of this is not clear. mRNA expression does not always correlate with protein expression. Therefore, further exploration of protein quantification is needed. This is perhaps because the main apical transporter is increased and results in a decrease in the other transporters that are not as necessary for apical transport. The current metabolomic data show an increase in kidney glucose and fructose which does not correlate with a decrease in transporter expression. It is likely that avian SGLTs have different substrate specificities and transport functions compared to the mammalian proteins.

In mammals, the SGLTs that are most important in glucose transport in the kidney are SGLT2 and, to a lesser extent, SGLT1. Chickens do not have an avian counterpart to SGLT2, which, in humans, is the most important transporter for glucose from the urine into the proximal tubule cells of the kidney [43]. Of the four SGLTs whose expressions we examined in the kidney, only SGLT4 showed increased expression in the LRWI birds. This may indicate that the SGLT responsible for apical glucose reabsorption in the chicken kidney is SGLT4 since they do not have SGLT2. This is not unprecedented as chickens are reported to also lack GLUT4 and likely use GLUT8 as their insulin-sensitive glucose transporter [44]. LRWI birds would then have increased expression of this transporter and increased reabsorption of glucose with water following. Again, this could reduce the need for water in the LRWI birds or it could be a response to lower water intake in these birds. In either case, increased mRNA expression of kidney SGLT4 is a marker for reduced water intake in broilers. However, further studies have to be conducted to confirm the utility of SGLT4 as the main apical glucose transporter in chickens.

## 5. Conclusions

The kidney plays a significant role in osmoregulation, excreting excess water or increasing water conservation when fluid intake is insufficient. Though much is known about how mammalian kidneys effect this control, less is known about avian kidneys. Our findings indicate that GLUT2 and SGLT4 are the main basal and apical transporters for glucose in broiler chickens, which have increased expression in LRWI birds. We therefore posit that SGLT4 is the apical glucose transporter which takes the place of the absent SGLT2 in chickens. It would therefore be responsible for the majority of the glucose reabsorption at the proximal tubule. Further experiments supporting SGLT4’s location in the kidney and further characterization should be undertaken. Also, further study of the molecular differences between HRWI and LRWI birds will advance our understanding of voluntary water intake and aid in mitigating the effects of climate change and water stress on our agricultural industries.

## Figures and Tables

**Table 1:** List of gene symbol, accession number, product size, and primer sequences used in qPCR.

Gene Symbol	Accession Number	Product Size	Primer Sequence
GLUT1	NM_205209.1	105 bp	Fwd 5′ CTTCTGCATACACTCCTTCTCC 3′
(SLC2A1)			Rev 5′ TGGACGTGAAACCAGCTAAA 3′
GLUT2	NM_207178.1	150 bp	Fwd 5′ TCATTGTAGCTGAGCTGTTCAGCC 3′
(SLC2A2)			Rev 5′ CGGCGAAGACAACGAACACATAC 3′
GLUT5	XM_417596.6	108 bp	Fwd 5′ AGGCTGATCTCTGCCTTTG 3′
(SLC2A5)			Rev 5′ GTCGATGTAGGTTCGGTTGTAG 3′
GLUT9	XM_420789.8	88 bp	Fwd 5′ GTGTCAGTCCTTCAGCTCCTTAGA 3′
(SLC2A9)			Rev 5′ CACAAAGCTGGTAGCATCCCATAG 3′
SGLT1	NM_001293240	97 bp	Fwd 5′ GAGGAGAAACCCGATGAAAGAG 3′
(SLC5A1)			Rev 5′ CTAAGCCACAGAACCAGTTGTA3 3′
SGLT4	XM_040678521.2	104 bp	Fwd 5′ GAGAGCATGACTTGGTCGAAAGTG 3′
(SLC5A9)			Rev 5′ GGACAAACCACAGAACCACAGATAC 3′
SGLT5	XM_046927938	89 bp	Fwd 5′ TGAGGGCTCAGGGCTCTTTAT 3′
(SLC5A10)			Rev 5′ CGTACGTCGCATTCCACTCAAA 3′
SGLT6	XM_414862.8	102 bp	Fwd 5′ GCTGCTACTTACGGTGGTCTCTAT 3′
(SLC5A11)			Rev 5′ TGCAGGTAGGAGCTGATGGATT 3′
βActin	NM_205518.2	125 bp	Fwd 5′ AGACATCAGGGTGTGATGGTTGGT 3′
			Rev 5′ TCCCAGTTGGTGACAATACCGTGT 3′

## Data Availability

Data are available upon reasonable request to the corresponding author.
